# Ghrelin-like peptide with fatty acid modification and *O*-glycosylation in the red stingray, *Dasyatis akajei*

**DOI:** 10.1186/1471-2091-10-30

**Published:** 2009-12-14

**Authors:** Hiroyuki Kaiya, Shiho Kodama, Koutaro Ishiguro, Kouhei Matsuda, Minoru Uchiyama, Mikiya Miyazato, Kenji Kangawa

**Affiliations:** 1 Biochemical research laboratories, ASUBIO PHARMA CO, LTD, 1-1-1, Wakayamadai, Shimamoto-cho, Mishima-gun, Osaka 618-8513, Japan; 2 Department of Biology, Faculty of Science, Toyama University, Toyama 930-8555, Japan; 3 Department of Biochemistry, National Cardiovascular Center Research Institute, Osaka 565-8565, Japan

## Abstract

**Background:**

Ghrelin (GRLN) is now known to be an appetite-stimulating and growth hormone (GH)-releasing peptide that is predominantly synthesized and secreted from the stomachs of various vertebrate species from fish to mammals. Here, we report a GRLN-like peptide (GRLN-LP) in a cartilaginous fish, the red stingray, *Dasyatis akajei*.

**Results:**

The purified peptide contains 16 amino acids (GVSFHPQPRS^10^TSKPSA), and the serine residue at position 3 is modified by *n*-octanoic acid. The modification is the characteristic of GRLN. The six N-terminal amino acid residues (GVSFHP) were identical to another elasmobranch shark GRLN-LP that was recently identified although it had low identity with other GRLN peptides. Therefore, we designated this peptide stingray GRLN-LP. Uniquely, stingray GRLN-LP was *O*-glycosylated with mucin-type glycan chains [*N*-acetyl hexosamine (HexNAc)_3 _hexose(Hex)_2_] at threonine at position 11 (Thr-11) or both serine at position 10 (Ser-10) and Thr-11. Removal of the glycan structure by *O*-glycanase made the *in vitro *activity of stingray GRLN-LP decreased when it was evaluated by the increase in intracellular Ca^2+ ^concentrations using a rat GHS-R1a-expressing cell line, suggesting that the glycan structure plays an important role for maintaining the activity of stingray GRLN-LP.

**Conclusions:**

This study reveals the structural diversity of GRLN and GRLN-LP in vertebrates.

## Background

Ghrelin (GRLN), which generally consists of 28 amino acids, was first identified in the stomachs of rats and humans as an endogenous ligand for the growth hormone secretagogue-receptor 1a (GHS-R1a)[1]. The serine residue at position 3 of this peptide (Ser-3) contains a unique octanoyl modification, and the acylation is necessary for the peptide to bind and activate GHS-R1a [1,2]. In mammals, GRLN is an important hormone involves in various physiological events such as pituitary, cardiovascular, steroidogenic, and developmental functions and energy homeostasis [3-6].

In non-mammals, GRLN has been identified in species from fish to birds (reviewed by [7-11]). Very recently, endogenous GRLN form was determined in a bony fish, goldfish [12]. Non-mammalian mature GRLNs are composed of 17 to 28 amino acids, and are known to involve in regulating pituitary functions in teleosts [6], amphibians [13] and birds [14], and feeding in teleosts [15-19] and birds [7,20-23]. An inhibitory effect of GRLN on drinking in birds (chicken) was also recently reported [24].

We identified a GRLN-like peptide (GRLN-LP) in the stomach of primitive vertebrates, cartilaginous fish, the hammerhead shark (*Sphyrna lewini*) and blacktip reef shark (*Carcharhinus melanopterus*) [25]. The GRLN-like peptide from both sharks consisted of 25 amino acids, and only three amino acids differed between the two species. Like other vertebrate GRLN, Ser-3 of GRLN-LP from sharks had been modified by *n*-octanoic or *n*-decanoic acid. However, the first seven N-terminal residues, which are generally highly conservative region of GRLN including the active core, had low identity between sharks (GVSFHPR) and those of other species (GSSFLSP, GTSFLSP and GSTFLSP). This difference is one of the reasons why this peptide was designated GRLN-LP though there is Ser-3 acylation. In addition to this, the C-terminal end of the shark GRLN-LP has not been amidated, which is a specific feature of teleost GRLN. However, we have proposed that shark GRLN-LP exhibits ancestral features of GRLN molecule that are present in higher vertebrate.

Here we report the structure of GRLN-LP from the stomachs of another elasmobranch, the red stingray, *Dasyatis akajei*, and identification of the cDNA that encodes the precursor protein. Interestingly, we revealed that the stingray GRLN-LP is not only octanoylated at Ser-3 but also possesses a mucin-type glycan structure at threonine (Thr)-11 or both Ser-10 and Thr-11.

## Methods

### Purification of GRLN-LP from stingray stomachs

Stingrays, *Dasyatis akajei*, were collected at Toyama bay (Toyama, Japan). Frozen stomachs (approximately 40 g) were used as the starting material. All animal experiments were conducted in accordance with Guidelines for the Care and Use of Animals of the University of Toyama and of National Cardiovascular Center (ref. no. 8053). GRLN was purified as previously described [26] with slight modifications. During the purification process, GRLN activity was monitored by measuring changes in the intracellular calcium ion (Ca^2+^) concentrations in a cell line that stably expressing rat GHS-R1a (CHO-GHSR62).

Stomach tissues were boiled in five volumes of Milli-Q level water, minced, acidified with concentrated acetic acid (AcOH) to 1 M, and homogenized. The supernatant was obtained by centrifugation. Next, cold acetone was added to the AcOH-extracts at a final concentration of 66%, after which the mixture was stirred over night and then centrifuged. The resulting supernatant was evaporated and purified with a Sep-Pak Vac 35 cc C18 cartridge (Waters, Milford, MA) to enrich the peptide components. The cartridge was successively eluted with 25% acetonitlile containing 0.1% trifluoroacetic acid (TFA) and 60% acetonitlile containing 0.1% TFA. The lyophilized Sep-Pak fraction that was eluted with 60% acetonitlile containing 0.1% TFA was dissolved in 1 M AcOH, and then subjected to cation-exchange chromatography using a SP-Sephadex C-25 (GE Healthcare UK Ltd., Buckinghamshire, England). Successive elution with 1 M AcOH, 2 M pyridine and 2 M pyridine-AcOH (pH 5.0) yielded three fractions: SP-I, SP-II and SP-III, respectively.

The basic peptide-enriched SP-III fraction was subjected to carboxymethyl (CM)-ion exchange HPLC (TSKgel CM-2SW, 4.6 × 250 mm, Tosoh, Tokyo, Japan) at a flow rate of 1 ml/min. A two-step gradient was made from solvent A (10 mM ammonium formate (pH 4.8) in 10% acetonitlile) to 25% solvent B (1 M ammonium formate (pH 4.8) in 10% acetonitlile) for 10 min and then to 55% solvent B for 90 min. The eluate was collected in 1-ml fractions from the start of the gradient program. Based on the assay results, GRLN activities were eluted in clusters. Thus, we performed secondary CM-HPLC on the active fractions with a more shallower two-step gradient profile from solvent A to 15% solvent B for 10 min and then to 35% solvent B for 80 min. The eluate was collected in 1-ml fractions every 1 min, 20 min after injection into the HPLC system.

Active CM-HPLC fractions were desalted by Sep-Pak treatment, lyophilized, and then subjected to an anti-rat GRLN1-11 immunoglobulin G (IgG) immuno-affinity column to purify GRLN-immuno-cross reactive substances [26]. This immuno-affinity column effectively absorbed the shark GRLN-LP [25]. The adsorbed substances were eluted with 60% acetonitlile containing 0.1% TFA and then separated by two different reverse-phase (RP)-HPLC methods. The samples were first applied to a preparative RP-HPLC with a Symmetry C18 column (3.9 × 150 mm, Waters) at a flow rate of 1 ml/min under a linear gradient from 10% to 60% acetonitlile containing 0.1% TFA for 40 min. Active fractions were further purified on a Symmetry C18 column (2.1 × 150 mm, Waters) or a diphenyl column (2.1 × 150 mm, 219TP5215, Vydac, Hesperia, CA) at a flow rate of 0.2 ml/min under a gradient from 10% to 60% acetonitlile containing 0.1% TFA for 40 min. The eluate that corresponded to each absorbance peak was collected. For peptide sequencing, approximately 5 pmol of the purified peptide, which was estimated based on the absorbance peak height, was subjected to protein sequencing (model 494, Applied Biosystems, Foster City, CA).

### Mass spectrometry

The molecular weights of the purified peptides were determined using matrix-assisted laser desorption ionization-time of flight-time of flight (MALDI-TOF-TOF) mass spectrometry (4700 proteomics analyzer, Applied Biosystems, Foster City, CA) with α-Cyano-4-hydroxycinnamic acid (CHCA) (Sigma-Aldrich Chem. Corp., WI) as a matrix.

### Prediction of *O*-glycosylation sites

The detected peptide mass and its profile indicated that the peptide was possibly glycosylated. The NetOGlyc program, which is available at the Center for Biological Sequences CBS Prediction Server http://www.cbs.dtu.dk, was used to predict the potential *O*-glycosylation sites.

### Cloning of stingray GRLN-LP cDNA

The nucleotide sequence of stingray GRLN-LP cDNA was determined using the Rapid Amplification of cDNA Ends (RACE) PCR Kit (Invitrogen, Carlsbad, CA). Total RNA (2 μg) was extracted from a stingray stomach and then reverse-transcribed using Omniscript RT (QIAGEN GmbH, Hilden, Germany). For 3'-RACE PCR, four degenerate primers were designed based on the sequence of the seven N-terminal amino acids in stingray GRLN-LP (G^1^VSFHPQ^7^) that was identified by protein sequencing. Among these primers, the rayGRL-s2 primer (5'-GGN GTN AGY TTY CAY CCN CA-3') effectively amplified expected cDNA fragment. PCR was performed using 50 pmol/reaction of rayGRL-s2, an adaptor primer supplied in the kit, *ExTaq *DNA polymerase (TaKaRa, Shiga, Japan) under the following amplification conditions: 94°C for 1 min, 35 cycles at 94°C for 30 sec, 53°C for 30 sec, and 72°C for 1 min; followed by a final extension for 3 min at 72°C. The amplified products were purified by the Wizard PCR Preps (Promega, Madison, WI). Second-round nested PCR was performed on the purified cDNA with four degenerate sense primers (50 pmol/reaction) that were based on the amino acid sequence of stingray GRLN-LP (F^4^HPQPRS^10^). Among these primers, two primers, rayGRL-s6 (5'-TTY CAY CCN CAR CCN CGN AG-3') and rayGRL-s8 (5'-TTY CAY CCN CAR CCN AGR AG-3') effectively amplified expected cDNA fragment under the following conditions: 94°C for 1 min, 35 cycles at 94°C for 30 sec, 55°C for 30 sec, and 72°C for 1 min; and a final extension for 3 min at 72°C. The approximately 400-bp amplified products were subcloned into the pCRII-TOPO vector (Invitrogen). The nucleotide sequence of the insert was determined by a DNA sequencer (ABI PRISM 3100 Genetic analyzer, Applied Biosystems), according to the BigDye^® ^Terminator Cycle Sequencing Kit protocol (Applied Biosystems) using the M13 forward or reverse primer.

For 5'-RACE PCR, two gene-specific primers were designed based on the partial sequence of the stingray GRLN-LP cDNA that was obtained by 3'-RACE PCR: rayGRL-as1, 5'-GGA CGA TGC ATT GAT CTG CGG-3' and ray GRL-as2, 5'-CCC GTT CAG GTC GGA CGA TGC-3'. Primary PCR was performed using rayGRL-as1, an anchor primer supplied in the 5'-RACE Kit, and *ExTaq *DNA polymerase under the following reaction conditions: 94°C for 2 min, 35 cycles at 94°C for 30 sec, 56°C for 30 sec, and 72°C for 1 min; and a final extension for 3 min at 72°C. The second-round nested PCR was performed with 5 pmol/reaction of the rayGRL-as2, an abridged universal amplification primer (AUAP) supplied in the 5'-RACE Kit, and *ExTaq *DNA polymerase under the same conditions described above. The rayGRL-as2 primer was designed inside the rayGRL-as1 primer, but nine nucleotides on the 3'-side of the rayGRL-as2 were identical to the primer sequence of rayGRL-as1. Thus, the target cDNA could be amplified effectively by the second-round nested PCR. The amplified products, which were approximately 480 bp, were subcloned into the pCRII-TOPO vector and sequenced.

### Amplification of the full-length stingray GRLN-LP cDNA

To confirm the precise nucleotide sequence of the full-length stingray GRLN-LP cDNA, we performed PCR using a proofreading, *Pyrobest *DNA polymerase (TaKaRa). The template cDNA for the 3'-RACE PCR was used because the Tm of the antisense primer for the 3'end of the stingray GRLN-LP cDNA was too low to amplify the full-length cDNA. Thus, PCR was conducted using a sense primer from the 5'end of the stingray GRLN-LP cDNA (5'-ACG ACC ACA GAT CCA ACT CGA-3') and AUAP under the following conditions: 98°C for 30 sec, 30 cycles at 98°C for 15 sec, 60°C for 30 sec, and 72°C for 1 min. For TA cloning, an additional 10-min cycle at 72°C was performed using *ExTaq *DNA polymerase (overhang reaction). The amplified product was subcloned into the pCRII-TOPO vector and sequenced.

The putative stingray GRLN-LP sequence was analyzed by BLAST against the NCBI database, and amino acid sequence alignment and identity analysis were performed by multiple comparison and maximum matching program using GENETYX-Mac ver. 15.0.1 (gap penalty, insert: -1; extend: -1 for multiple comparison, and default condition for maximum matching). Phylogenetic tree was made using Mega 4 software http://www.megasoftware.net/.

### Deglycosylation with *O*-glycanase

Stingray GRLN-LP was *O*-glycosylated with mucin-type sugar chains. To examine the role of this sugar chain modification in the GRLN-like activity of stingray GRLN-LP, a high-yield preparation of purified stingray GRLN-LP (peak 2, Table 1) was treated with the *O*-glycanase, end-α-*N*-acetylgalactosaminidase (Calbiochem, Darmstadt, Germany). This enzyme catalyzes the hydrolysis of the unsubstituted Galβ1,3GalNAc core dissaccharide attached to a Ser or Thr of glycopeptides to generate free oligosaccharides. Approximately 5 pmol of native stingray GRLN-LP (peak 2), synthesized stingray des-acyl GRLN-LP, and octanoylated stingray GRLN-LP were incubated separately with 1.25 mU *O*-glycanase in 100 μl 50 mM sodium phosphate (NaH_2_PO_3_) buffer (pH 5.0) for 16 h at 37°C, followed by an incubation for 15 min at 70°C to inactivate the enzyme. The reaction mixture was subjected to RP-HPLC on a Symmetry C18 column (2.1 × 150 mm, Waters) at a flow rate of 0.2 ml/min under a linear gradient from 10% to 60% acetonitlile containing 0.1% TFA for 40 min. The absorbance peaks that corresponded to each catalyzed peptide were collected. Two peaks appeared from this reaction: one was stingray GRLN-LP without sugar chains, and the other was the original native peptide. We did not observe any changes in the elution pattern of synthetic stingray des-acyl GRLN-LP and octanoylated GRLN-LP after the catalytic treatment.

**Table 1:** Summary of stingray GRLN-LP purification

Group	Peak	Yields (pmol)	Mass-1 [M+H]^+^	Mass-2 [M+H]^+^
A	1	10	2580.15	
B	2	24	3351.68	3513.74
	3	4	3375.42	3537.46
C	4	21	2580.26	2742.33
	5	4	2604.15	2766.20
	6	3	2604.17	2766.26
D	7	2	2594.15	2756.21

### Quantitative real-time PCR of stingray GRLN-LP in stingray tissues

We performed quantitative real-time PCR (qPCR) to examine the GRLN-LP cDNA expression levels in various stingray tissues. Total RNA was extracted from 21 tissues from two separate stingrays to examine the variation in expression levels. First-strand cDNAs were synthesized from 1 μg of DNase-I- (Invitrogen) treated total RNA using the QuantiTect RT Kit (QIAGEN GmbH) and an oligo-dT_12-18 _primer. PCR amplification was performed using the LightCycler system (Roche Applied Science, Mannheim, Germany) and the QuantiFast SYBR Green PCR Kit (QIAGEN GmbH). To generate a standard curve, full-length stingray GRLN-LP cDNA or partial stingray β-actin cDNA fragment in the pCRII-TOPO vector was linearized by *Xba*-I digestion. The linearized plasmid was serially diluted from 5 × 10^6 ^to 5 × 10^3 ^molecules to generate a standard curve that was used to determine the cDNA copy number. All qPCR amplifications were performed in duplicate. All specific quantities were normalized as the copy number relative to the total RNA [27] and to stingray β-actin. For the GRLN-LP analysis, sense (5'-TCC CTC ACC CTC AAG GCA GAG-3') and antisense primers (5'-TCAT CTC CCA CTG GCA ACT GG-3') were designed to amplify a 170-bp product. For the β-actin analysis, sense (5'-GAT CTG TAT GCC AAC AAC GTC-3') and antisense primers (5'-CAG AGA TGC CAG AAT AGA GCC-3') were designed to amplify a 194-bp product. The PCR reaction mixture (20 μl) contained 100 ng of cDNA, 1× QuantiFast SYBR Green PCR mix, and 5 pmol of each primer was prepared according to the manufacturer's protocol. The PCR conditions were 95°C for 5 min, 35 cycles at 95°C for 10 sec, 60°C for 30 sec. Amplified products were electrophoresed on a 1.5% agarose gel to determine the product size and quantity.

## Results

### Purification of stingray GRLN-like peptide

**Figure 1:**
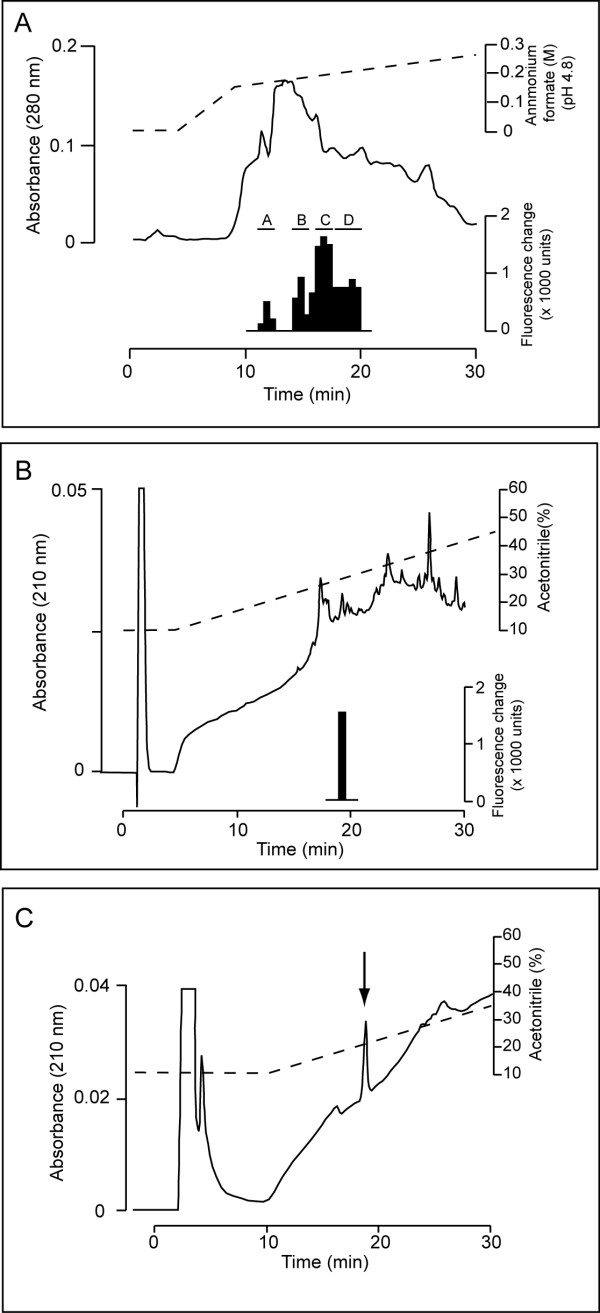
**Purification of stingray ghrelin-like peptide (GRLN-LP) from stomach extracts**. *Black bars *indicate the measured fluorescence changes in intracellular calcium ion concentrations in CHO cells expressing rat GHS-R1a (CHO-GHSR62). (A) Carboxymethyl (CM)-cation ion-exchange HPLC (pH 4.8) of the SP-III fraction of stomach extracts. The GRLN-like activity was divided into four groups (A-D). (B) Preparative reverse-phase (RP)-HPLC (Symmetry C18, 3.9 × 150 mm) of group B after purification with an anti-rat GRLN1-11 immuno-affinity column. (C) Final purification of the active fraction indicated in (B) by another RP-HPLC (Vydac diphenyl, 219TP5215, 2.1 × 150 mm).

The SP-III fraction from the SP-Sephadex C-25 cation-exchange chromatography contained GRLN-like activity. Next, we subjected the basic peptide-enriched SP-III fraction to CM-HPLC (pH 4.8) with a gradient program. However, the GRLN-like activities are present in positions where a large number of peptides eluted (fractions 17-29, data not shown). To reduce the peptide content, active fractions were subjected again to CM-HPLC with a much shallower two-step gradient profile. As a result, the GRLN-like activity was divided into four groups from A to D (Fig. 1A). Each group was purified by anti-rat GRLN1-11 IgG immuno-affinity column chromatography, followed by RP-HPLC until a single peak was isolated. Figs. 1B and 1C show representative preparative RP-HPLC and final RP-HPLC profiles after immuno-affinity chromatography of group B (Fig. 1A).

The estimated peptide yield from each peak height is summarized in Table 1. Overall, seven species of peptides were isolated. The amino acid sequences of peptides in high yield peaks 2 and 4 were analyzed. The amino acid sequences of 16 residues were determined: GVXFHPQPRXXSKPSA for peak 2 and GVXFHPQPRSXSKPSA for peak 4 (X, unidentified). The amino acid at position 3 was not detected, probably due to the characteristic acyl modification at this position of GRLN. However, the reason why amino acid at positions 10 and 11 could not be identified was uncertain.

### Cloning of cDNA encoding the stingray peptide precursor

**Figure 2:**
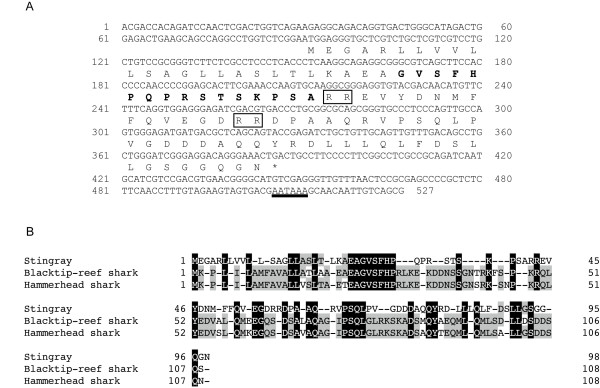
**Nucleotide sequences and deduced amino acid sequence of stingray GRLN-LP**. (A) Nucleotide sequence and deduced amino acid sequence of stingray GRLN-LP. The cDNAs have been deposited in the DDBJ/EMBL/GenBank™ databases (AB480033). *Bolded letters *indicate isolated peptide. A typical processing signal (RR) is *boxed*, and the polyadenylation signal (AATAAA) is *underlined*. (B) Comparison of the amino acid sequences of GRLN-LP in elasmobranch. Sequence alignment was performed using GENETYX-Mac ver 15.0.1. The sequences that are identical to all species are densely shadowed, and the sequences conserved in more than two species are thinly shadowed.

**Figure 3:**
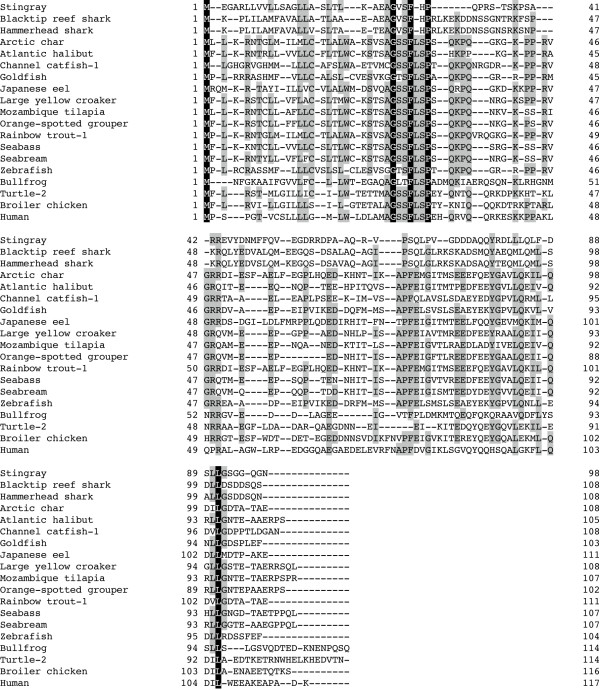
**Multiple comparisons of amino acid sequence of stingray GRLN-LP**. Sequence alignment was performed using GENETYX-Mac ver 15.0.1. Amino acids that are identical to all species are densely shadowed, and amino acids conserved in more than two species are thinly shadowed.

**Figure 4:**
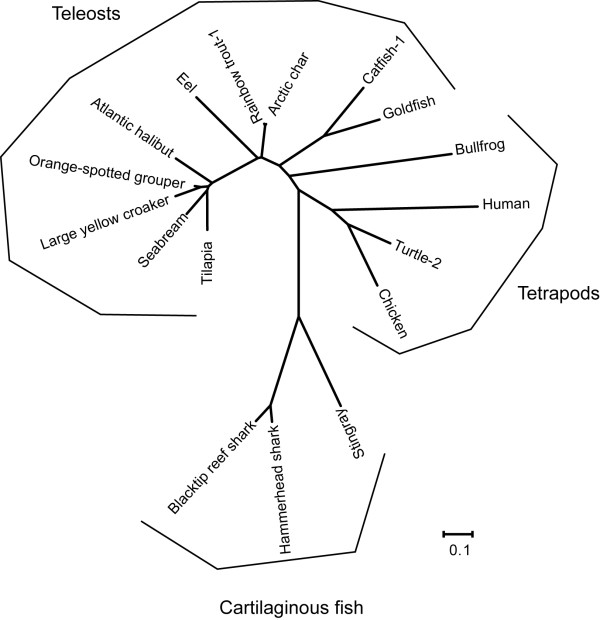
**Phylogenetic analysis of stingray GRLN-LP**. Phylogenetic tree was generated using the neighbour-joining method by MEGA4 http://www.megasoftware.net/(Tamura et al., 2007). Amino acid sequences are available from the DDBJ/EMBL/GenBank databases as shown in Table 2.

To determine the complete amino acid sequence of the stingray peptide, we isolated cDNA encoding the peptide precursor from stingray stomach mRNA based on the identified amino acid sequence. The identified nucleotide sequence of the stingray peptide was 527 bp in length, which consisted of a 90-bp 5' untranslated region (UTR), a 297-bp coding region, and a 140-bp 3'UTR (accession number AB480033, Fig. 2A). A polyadenylation signal was present in the 3'UTR (positions 506-511). The deduced amino acid sequence showed that the stingray peptide precursor consisted of 98 amino acids. The unidentified amino acids at positions 3, 10 and 11 were identified as Ser-3, Ser-10 and Thr-11, respectively. Therefore, this newly identified stingray peptide contains 16 amino acids, GVSFHPQPRSTSKPSA. A typical dibasic processing signal (arginine-arginine, RR) followed the peptide, and processing at this site generated a peptide that was identical to the purified peptide. In addition, we identified another dibasic processing signal 14-amino acids downstream of the first processing sequence (Fig. 2A). Based on this motif, it is expected that a 31-amino acid peptide could be generated. However, this 31-amino acid peptide was not identified during this purification process. Deduced stingray peptide precursor showed relatively high sequence identities compared with shark GRLN-LP precursors (Fig. 2B, Table 2). Multiple comparisons of the precursor protein revealed that the identity of the stingray peptide was low compared to GRLNs from teleosts to mammals (Fig. 3, Table 2). Fig. 4 shows a phylogenetic tree of GRLN, and the stingray peptide was categorized in the same clade as shark GRLN-LP.

**Table 2:** Amino acid sequence identity of stingray preproGRLN-LP with other preproGRLNs

Species	Identity (%)	Accession No.
Arctic char	21.1	AB490668
Atlantic halibut	23.3	EF493849
Blacktip-reef shark	34.5	AB254129
Broiler chicken	26.9	AB075215
Bullfrog	26.3	AB058510
Channel catfish-1	26.8	AB196449
Goldfish	25.9	AF454389
Hammerhead shark	32.7	AB254128
Human	25.8	AB029434
Japanese eel	19.8	AB062427
Large yellow croaker	27.0	FJ560488
Mozambique tilapia	22.9	AB077764
Orange-spotted grouper	23.6	DQ343147
Rainbow trout-1	20.7	AB096919
Sea bass	22.4	DQ665912
Seabream (Black porgy)	20.6	AY643808
Turtle-2	21.4	AB161458
Zebrafish	25.2	NM_001083872

### Mass spectrometric analyses of the purified stingray peptide

**Figure 5:**
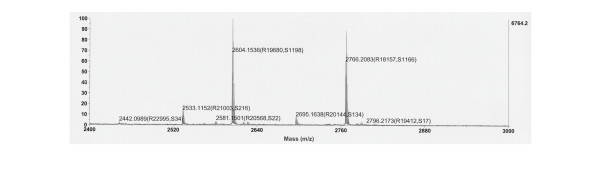
**MALDI-TOF mass spectrum of an isolated stingray GRLN-LP**. Peak 5 peptide, which is indicated in Table 1, was analyzed.

The protein sequencing results strongly indicated that Ser-3 was acylated. The theoretical mono-isotopic mass of a 16-residue peptide with *n*-octanoic acid modification is 1808.87 [M+H]^+^. During the analysis, we observed two types of mass peak profiles; one profile showed a single peak, while the other exhibited two peaks that had a constant mass difference of 162.1 (Fig. 5). The pattern of this spectrum is characteristic of glycosylated proteins or peptides, and the mass difference corresponded to the mass of hexose (Hex). The mass values measured for the peptides were greater from 795.3 to 957.3 than the predicted mass for an octanoylated 16-amino acid peptide (Table 1). Therefore, we assumed that all purified peptides were glycosylated.

**Figure 6:**
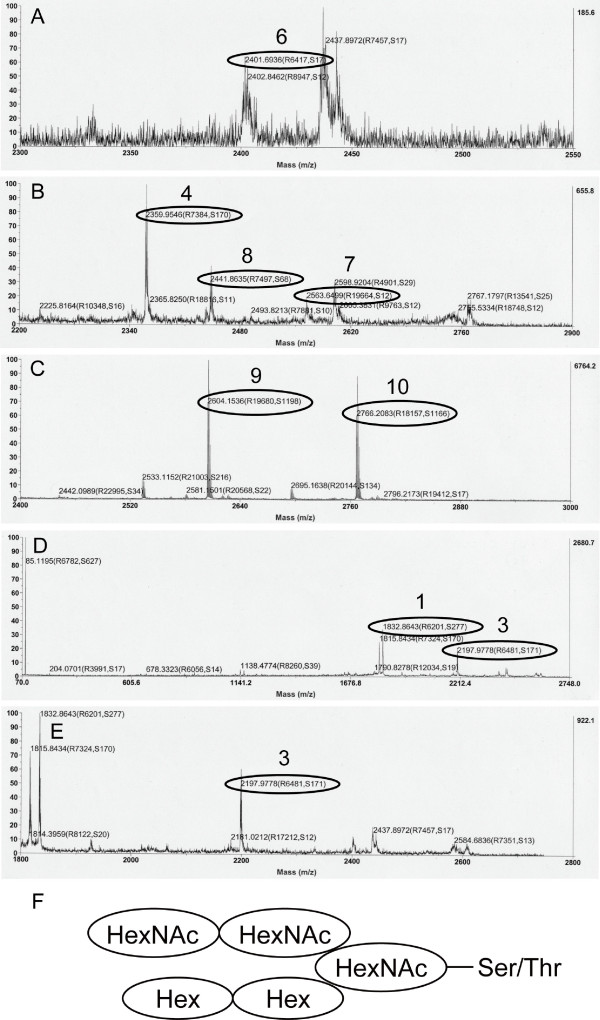
**MALDI-TOF MS/MS spectra of an isolated stingray GRLN-LP**. Peak 5 peptide (parent mass of m/z 2766.2), which is indicated in Table 1, was analyzed by MALDI-TOF MS/MS spectrometry. The glycan chains that are predicted from the fragment masses (A-E) are shown in Table 3. (F) Predicted structure of the glycan chains with a type-2 core.

**Figure 7:**
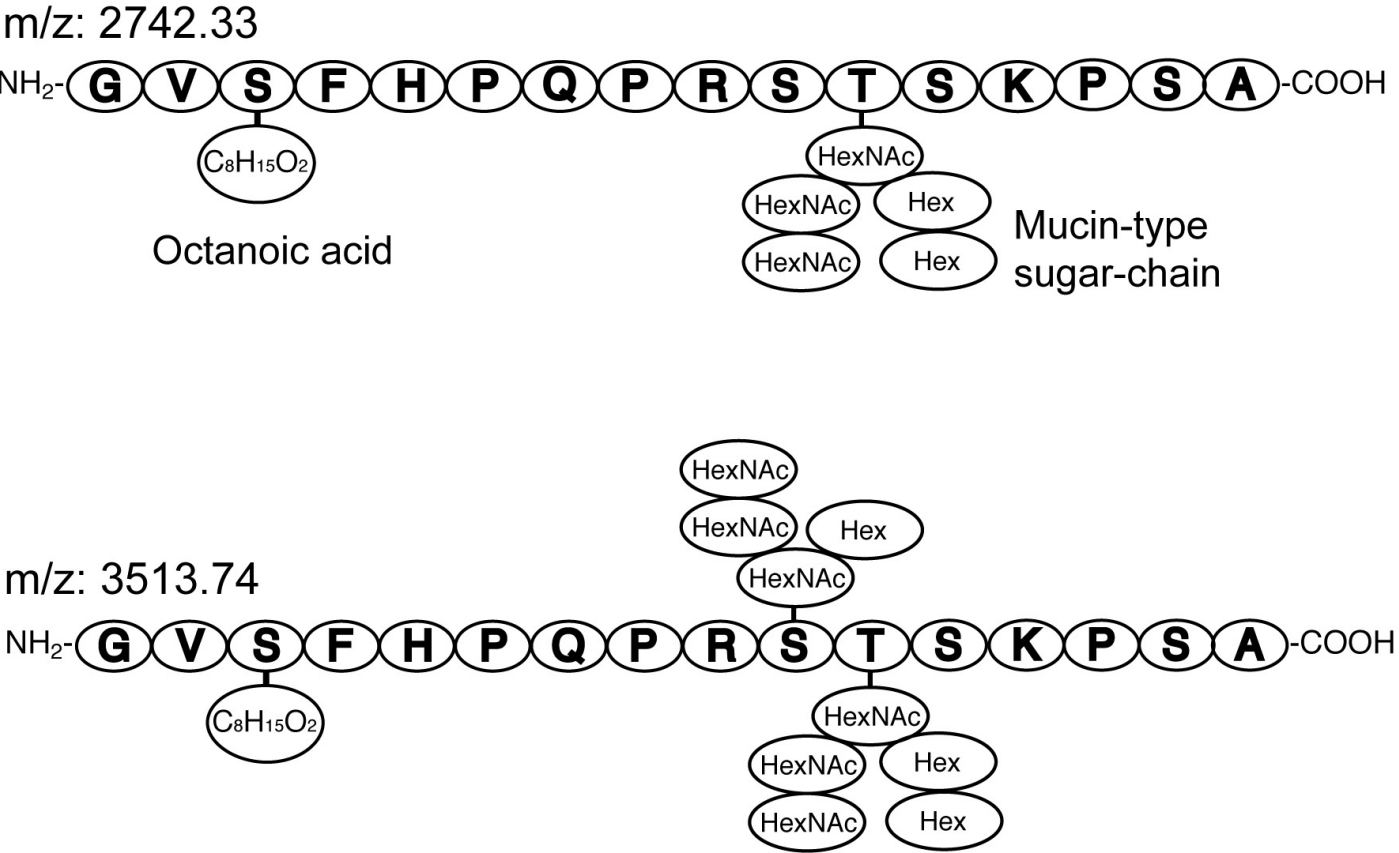
**Predicted primary structures of stingray GRLN-LP**. Stingray GRLN-LP is modified by *n*-octanoic acid at Ser-3, and mucin-type sugar chains at Thr-11 or both Ser-10 and Thr-11. These structures were predicted based on protein sequencing and MALDI-TOF MS/MS of peak 4 (mass-2, m/z 2742.33) and peak 2 (mass-2, m/z 3513.74) that are indicated in Table 1. The predicted compositions of the glycan chains of other isolated stingray GRLN-LPs are shown in Table 4.

We analyzed the potential glycosylation sites in the identified peptide sequence using the NetOGlyc program, and found that Thr-11 is a potential *O*-glycosylation site (G-score 0.728, I-score 0.521). Interestingly, Thr-11 corresponded to one of the unidentified amino acids in the protein sequencing. The most common type of *O*-glycosidic linkage is an attachment through the *N*-acetyl hexosamine (HexNAc) to the side chain of Ser or Thr. Thus, we analyzed peak 3 (parent masses of m/z 2580.26 and 2742.33) using MALDI-TOF MS/MS spectrometry. As a result, we were able to assign a mass to the octanoylated stingray peptide (1808.8 [M+H]^+^) from each parent mass (data not shown), indicating that the peak 3 stingray peptide is octanoylated. In addition, glycoside and glycochain-specific masses, Hex (162), HexNAc (203) and HexNAc-Hex (365) were assigned based on an MS/MS analysis of the parent mass of m/z 2580.26, and HexNAc3-Hex1 (771) and HexNAc3-Hex2 (933) were identified from an MS/MS analysis of the parent mass of m/z 2742.33 (data not shown). Since one unit of HexNAc-Hex was assigned, it was predicted that this glycochain might have a mucin-type core-1 or core-2 structure. Based on this information, we next attempted to identify the composition of the mucin-type glycan chains using a purified peptide from peak 5 (parent mass of m/z 2766.20). The fragment masses that were estimated based on the expected glycan chains are shown in Table 3. In this analysis, the peak observed at m/z 1832.8 was m/z 24 greater than the mass of octanoylated 16-amino acid peptide. We could not be identified what is the origin of m/z 24. Fig. 6 shows the fragment mass spectra obtained from an MS/MS analysis of the parent mass. Each fragment mass shown in Table 3 was assigned as shown in Figs. 6A to 6E. Finally, the structure of the mucin-type glycan chains was predicted as shown in Fig. 6F. Representative predicted primary structures of the stingray peptide are shown in Fig. 7. A mass difference of 162.1 is considered to occur when one Hex is deleted from the glycan chain, suggesting that the mass-2 shown in Table 1 is the molecular mass of the original form. Table 4 summarizes the predicted compositions of the glycan chains in the isolated stingray peptide. The mass spectrometric analysis showed that all purified peptides would be modified by *n*-octanoic acid. Based on the high identity with shark GRLN-LP and the octanoyl modification of this peptide, we named this peptide stingray GRLN-LP.

**Table 3:** Expected mass spectra detected from GRLN-LP modified by glycan chains

No.	Peptide	Predicted glycan chains	Sum of glycan chains	Expected mass
1	GRLN-LP			1832.8*
2		HexNAc1	203.07	2035.87
3		HexNAc1-Hex	365.12	2197.92
4		HexNAc1-Hex2	527.17	2359.97
5		HexNAc2	406.14	2238.94
6		HexNAc2-Hex	568.19	2400.99
7		HexNAc2-Hex2	730.24	2563.04
8		HexNAc3	609.21	2442.01
9		HexNAc3-Hex	771.26	2604.06
10		HexNAc3-Hex2	933.31	2766.11

Note: * Detected mass is GRLN-LP that was modified by octanoic acid (*m/z *1808.8 [M+H]^+^) containing unidentifed adduct (*m/z *24).

**Table 4:** Predicted compositions of glycan chains in isolated stingray GRLN-LP

Peak	Mass [M+H]^+^	Possible compositions of glycan chains	Remarks
1	2580.15	Thr-11+(HexNAc)3(Hex)	Single peak
2	3351.68	Ser-10+(HexNAc)3(Hex)	m/z 2580.14 was detected
		Thr-11+(HexNAc)3(Hex)	
	3513.74	Ser-10+(HexNAc)3(Hex)	m/z 2580.14 was detected
		Thr-11+(HexNAc)3(Hex)2	
3	3375.42	Ser-10+(HexNAc)3(Hex)	m/z 24 adduct of peak 2
		Thr-11+(HexNAc)3(Hex)	
	3537.46	Ser-10+(HexNAc)3(Hex)	m/z 24 adduct of peak 2
		Thr-11+(HexNAc)3(Hex)2	
4	2580.26	Thr-11+(HexNAc)3(Hex)	
	2742.33	Thr-11+(HexNAc)3(Hex)2	
5, 6	2604.15	Thr-11+(HexNAc)3(Hex)	m/z 24 adduct of peak 1
	2766.20	Thr-11+(HexNAc)3(Hex)2	m/z 24 adduct of peak 1
7	2594.15	Thr-11+(HexNAc)3(Hex)	m/z 14 adduct of peak 4
	2756.21	Thr-11+(HexNAc)3(Hex)2	m/z 14 adduct of peak 4

### Tissue expression of GRLN-LP mRNA in stingray

**Figure 8:**
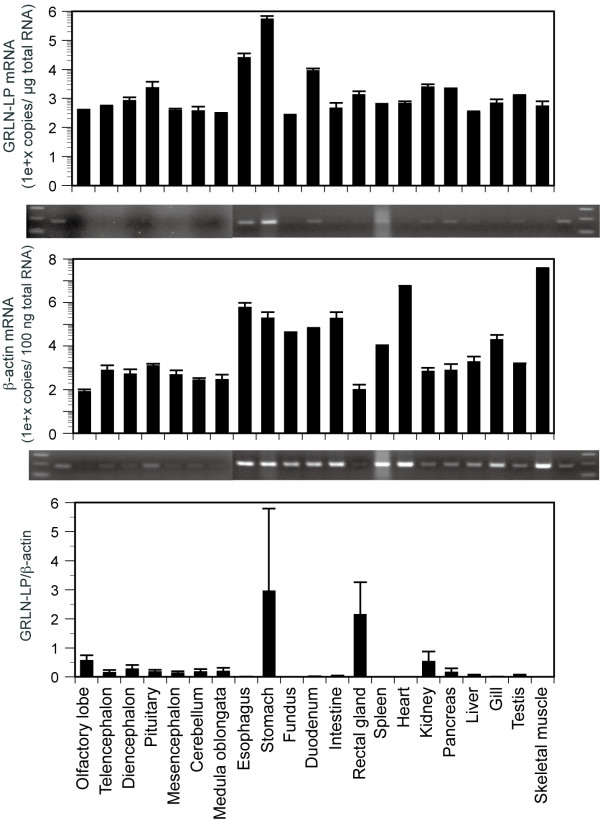
**Tissue expression pattern of GRLN-LP mRNA in the red stingray**. Quantitative real-time PCR results of GRLN-LP (top) and for β-actin (middle) are shown. Ratio of GRLN-LP to the β-actin gene is shown in the bottom. The values represent the means (± SD) of two different stingrays. Representative gel views obtained after 30 amplification cycles are displayed under each graph. The first and last two lanes contain the 100-bp ladder and the positive control in which PCR product of 1000-copies plasmid DNA standard finished by 35 amplification cycles.

Stingray GRLN-LP mRNA is expressed in almost of all of the examined tissues. The highest expression levels were in the stomach, with moderate levels in the pituitary, esophagus and duodenum (Fig. 8, top). Expression level of the β-actin gene varied among tissues (Fig. 8, middle): muscle tissues highly express the β-actin gene compared to soft tissues such as brain. Ratio of GRLN-LP mRNA to the β-actin gene was the highest in the stomach, followed by the rectal gland (Fig. 8, bottom).

### Biological activity of stingray GRLN-LP

**Figure 9:**
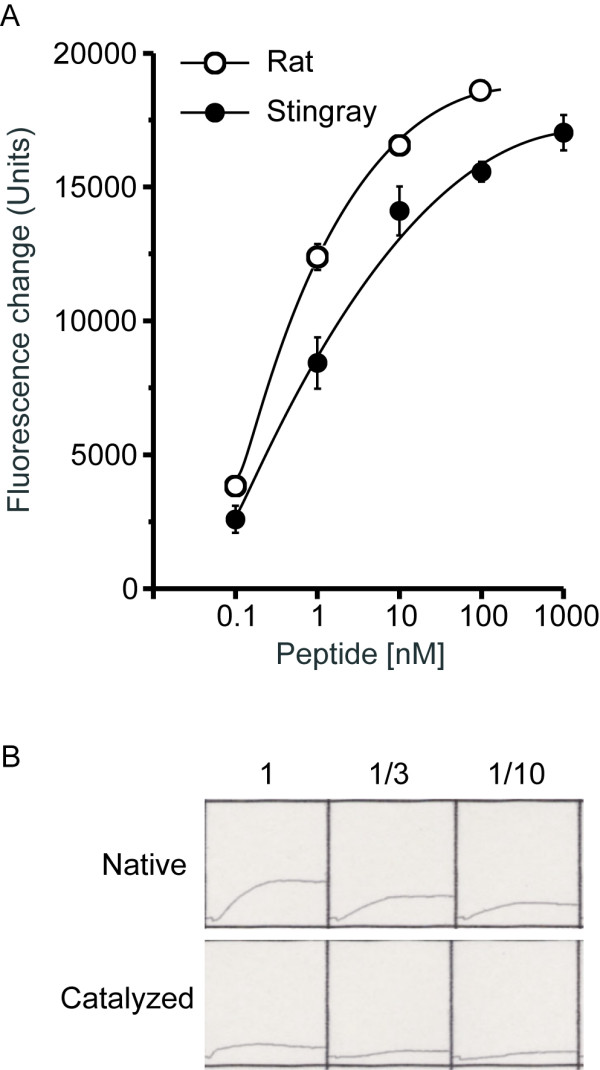
**GRLN-like activity of stingray GRLN-LP**. (A) Dose-response effects of rat GRLN (open circle) and unglycosylated stingray GRLN-LP (closed circle) on intracellular Ca^2+ ^concentrations in rat GHS-R1a-expressing cell line. Values represent the means (± SE) of samples tested in triplicate. (B) Reduction of GRLN-like activity after the native stingray GRLN-LP was catalyzed. The changes in intracellular Ca^2+ ^levels are shown for both the native (upper) and catalyzed (lower) peptides. The increases in intracellular Ca^2+^concentrations decreased after the native stingray GRLN-LP was catalyzed by *O*-glycanase. A dose response was obtained by diluting the original sample 1/3 and 1/10.

The unglycosylated synthetic stingray GRLN-LP dose-dependently increased intracellular Ca^2+ ^levels in CHO-GHSR62 cells stably expressing rat GHS-R1a (Fig. 9A). Compared to the homologous ligand, rat GRLN, the dose-response curve for the stingray peptide was shifted to the right, suggesting that the stingray peptide has a lower affinity for rat GHS-R1a than rat GRLN. Catalysis of the *O*-glycosylation of the native (glycosylated) stingray GHRL-LP reduced this activity (Fig. 9B, bottom).

## Discussion

In the present study, we identified a 16-amino acid peptide that exhibits GRLN-like activity in the stomach of an elasmobranch, the red stingray. Only a few peptide hormones have been identified in the stingray, notably calcitonin [28], relaxin-like molecule [29], pituitary adenylate cyclase-activating polypeptide (PACAP) [30] and proopiomelanocortin [31]. The result of the present study would become one of valuable information on peptide that exists in stingray.

GRLN is characterized by the modification of Ser-3 with fatty acids such as *n*-octanoic or *n*-decanoic acid in both mammals and non-mammals [3,8]. This acylation is essential for GRLN to bind to GHS-R1a and elicit GRLN activity [2]. Isolated stingray peptides activated a rat GHS-R1a-expressing cell line, suggesting that all peptides have GRLN-like structures, and the amino acid at position 3 may be acylated. In fact, peptide sequence and mass spectrometric analyses revealed that amino acid sequences of isolated stingray peptides are similar to shark GRLN-LP [25], and the isolated peptides were octanoylated. These data strongly suggest that the identified peptides are stingray GRLN or GRLN-LP.

We have identified GRLN-LP in another elasmobranch shark [25]. The peptide was named GRLN-LP because it had the following characteristics: (1) rat GHS-R1a activation properties, (2) presence of acyl-modifications at Ser-3, (3) predominant mRNA expression in the stomach and (4) similar gene organization compared to the GRLN gene. The newly identified stingray peptide satisfies three of these four structural features with an exception (4). Furthermore, the six N-terminal amino acids (GVSFHP) are identical between shark and stingray. In a phylogenetic analysis of the precursor protein, the stingray peptide and shark GRLN-LPs belong to the same clade. Although it is necessary to confirm that the stingray peptide possesses the characteristic GRLN activities such as stimulation of GH release and hyperphagia using a stingray or rodent model, we designate this peptide stingray GRLN-LP. Strictly, all structures, including glycosylation as mentioned below, are present in stingray GRLN-LP. It is interesting to note that mechanisms governing the acylation of Ser-3 are conserved from elasmobranches to mammals. Recently an acyltransferase, namely ghrelin-*O*-acyltransferase (GOAT), was identified [32,33], although the detailed mechanisms of acylation are still unknown [34,35].

Stingray GRLN-LP possessed *O*-glycosylation at Ser-10 and Thr-11, in addition to the acyl-modification at Ser-3. This glycosylation has not been seen in other GRLN/GRLN-LP so far. To date, little is known about the presence of *O*-glycosylated peptide hormones other than glycoproteins or peptides such as gonadotropins, prohormone, and vespulakinins (a wasp venom) [36-39]. These glycosylation positions of stingray GRLN-LP were found by using the NetOGlyc program http://www.cbs.dtu.dk, suggesting a glycosylation motif may be present. Mass spectrometric analyses of purified peptides revealed that stingray GRLN-LP possesses an elongated glycan structure based on a type-2 core (Figs. 6F and 7). For the formation of this glycan, β-1,3-galactose transferase and β-1,6-*N*-acetylglucosamine-transferase would be involved in the elongation. Glycosylation, as well as acylation by GOAT [32,33], is a post-translational modification. Further studies are required to determine if these transferases and GOAT co-localize in GRLN-LP-producing cells in the stingray stomach.

The functions of carbohydrates that are attached to proteins range from effects on protein folding to the formation of antigen-recognition sites. The functional significance of the glycosylation of stingray GRLN-LP is still unknown. This modification is likely involved in maintaining the biological activity of GRLN-LP because the native stingray GRLN-LP deglycosylating by *O*-glycanase resulted in a decrease in intracellular Ca^2+ ^concentrations in rat GHS-R1a-expressing cells (Fig. 8B). However, the glycosylation was not necessary to exhibit GRLN-like activity because unglycosylated synthetic stingray GRLN-LP could activate a rat GHS-R1a-expressing cell line (Fig. 8A). Furthermore, the C-terminal portion of GRLN, except the seven N-terminal amino acids, is important to maintain GRLN activity in the circulation. Consistent with these findings, truncated GRLN molecules with portions of the C-terminus deleted, do not stimulate GH release in rats *in vivo *[40]. The C-terminal portion of GRLN is rich in basic amino acid residues such as arginine (Arg), lysine (Lys) and histidine, *e.g.*, eight residues in human GRLN8-28 and five in eel GRLN8-21. On the other hand, stingray GRLN-LP has only two basic amino acids, Arg-9 and Lys-13. It is speculated that the mucin-type glycan chains may regulate the physicochemical features of this peptide and act as polar molecules that stabilize GRLN-LP in the circulation of the stingray. Further studies are required to understand the functional relevance of glycosylation of stingray GRLN-LP, such as determining the feeding-stimulatory or GH-releasing activity of glycosylated or deglycosylated GRLN-LP after injection into the stingray circulation *in vivo*.

GRLN-LP mRNA was predominantly expressed in the stomach. This expression pattern is consistent with that of GRLN in other vertebrates. GLRN has multiple physiological functions such as regulation of GH release, appetite regulation, cardiovascular function, gastrointestinal motility, pancreatic function and reproductive function from fish to mammals as endocrine/paracrine factors [3-6,8]. GRLN-LP could play some roles in homeostatic regulation in the stingray. However, physiological functions of GRLN-LP in the stingray have been still undetermined at present. It is interesting to note that Caminos et al. [41] reported that GRLN has a role in cartilage metabolism in humans, mice and rats. This could be a dominant effect in cartilaginous fish such as stingray. Further research is necessary to clarify physiological roles of GRLN-LP in the stingray.

Elasmobranchii is a species that have primitive characteristics of vertebrates. It is interesting to note that the GRLN-LP of the red stingray has more complicated post-translational modifications. We have determined GRLN-LP in two elasmobranch sharks, hammerhead shark and blacktip reef shark [25], and their GRLN-LPs do not have the glycosyl modification. Which is more primitive form of GRLN-LP? Chondrichthyes include two subclasses, the Elasmobranchii and the Holocephali [42]. Stingrays and sharks belong to the formers, and it is divided roughly as superorders, Batoidea and Selachimorpha, respectively. Hammerhead and blacktip reef shark, and stingray belong to the order Carcharhiniformes and Rajiformes, respectively. It has been considered that Rajiformes evolved later than Carcharhiniformes [43,44]. The modification of *O*-glycosylation seems to be specific to the red stingray for two reasons: (a) both the Ser-10 and Thr-11 are not evolutionarily conserved; and (b) the more ancient GRLN-LP in shark has no *O*-glycosylation. Based on this division, shark GRLN-LP might exhibit a more primitive structure, and the stingray GRLN-LP structure may be an advanced form.

Lastly, it has been reported that a novel appetite-inhibiting hormone, obestatin, is present in the C-terminal peptide of the GRLN precursor in mammals [45]. In this study, the potential processing signals that would release obestatin were not found in the stingray GRLN-LP precursor as well as in sharks [25], suggesting that obestatin-like peptide does not exist in the stingray from the structural view point.

## Conclusions

We characterized both the structure and tissue expression of GRLN-LP in a primitive vertebrate (cartilaginous fish), the red stingray. The structure of stingray GRLN-LP is very unique and is modified by both a mucin-type sugar chain and an *n*-octanoyl modification, indicating that stingray GRLN-LP reflects an ancestral form of the GRLN molecule. However, we are unable to conclude definitively that this peptide is GRLN because the characteristic biological properties of GRLN peptides, such as GH-releasing activity or appetite-stimulating activity, have not been confirmed for this peptide. Since the unglycosylated synthetic stingray GRLN-LP activated rat GHS-R1a-expressing cells in an *in vitro *assay system, further studies are necessary to elucidate the physiological functions of GRLN-LP in the stingray as well as determining the functional importance of the glycosylation of this peptide.

## Authors' contributions

HK performed almost all of the experiments, such as peptide purification, cDNA cloning, real-time PCR, phylogenetic analysis, functional analysis of the peptide, and drafted the manuscript. SK participated in the structural analyses of the glycan chains. KM, KI and MU extracted the stingray tissues and isolated the tissue RNA. MM and KK participated in the design and coordination of this study and helped draft the manuscript. All authors read and approved the final manuscript.

## References

[B1] Kojima M Hosoda H Date Y Nakazato M Matsuo H Kangawa K Ghrelin is a growth-hormone-releasing acylated peptide from stomach Nature 1999 402 656 660 10.1038/45230 10604470

[B2] Muccioli G Papotti M Locatelli V Ghigo E Deghenghi R Binding of ^125^I-labeled ghrelin to membranes from human hypothalamus and pituitary gland J Endocrinol Invest 2001 24 RC7 9 11314756 10.1007/BF03343831

[B3] Kojima M Kangawa K Ghrelin: structure and function Physiol Rev 2005 85 495 522 10.1152/physrev.00012.2004 15788704

[B4] Hosoda H Kojima M Kangawa K Biological, physiological and pharmacological aspects of ghrelin J Pharmacol Sci 2006 100 398 410 10.1254/jphs.CRJ06002X 16612045

[B5] Soares JB Leite-Moreira AF Ghrelin, des-acyl ghrelin and obestatin: Three pieces of the same puzzle Peptides 2008 29 1255 1270 10.1016/j.peptides.2008.02.018 18396350

[B6] Isgaard J Barlind A Johansson I Cardiovascular effects of ghrelin and growth hormone secretagogues Cardiovasc Hematol Disord Drug Targets 2008 8 133 137 10.2174/187152908784533676 18537600

[B7] Unniappan S Peter RE Structure, distribution and physiological functions of ghrelin in fish Comp Biochem Physiol A 2005 140 396 408 10.1016/j.cbpb.2005.02.011 15936698

[B8] Kaiya H Darras VM Kangawa K Ghrelin in birds: its structure, distribution and function J Poult Sci 2007 44 1 18 10.2141/jpsa.44.1

[B9] Kaiya H Miyazato M Kangawa K Peter RE Unniappan S Ghrelin: a multifunctional hormone in non-mammalian vertebrates Comp Biochem Physiol A 2008 149 109 28 10.1016/j.cbpa.2007.12.004 18222718

[B10] Yeung CM Chan CB Woo NY Cheng CH Seabream ghrelin: cDNA cloning, genomic organization and promoter studies J Endocrinol 2006 189 365 379 10.1677/joe.1.06593 16648303

[B11] Li X He J Hu W Yin Z The essential role of endogenous ghrelin in growth hormone expression during zebrafish adenohypophysis development Endocrinology 2009 150 2767 2774 10.1210/en.2008-1398 19264876

[B12] Miura T Maruyama K Kaiya H Miyazato M Kangawa K Uchiyama M Shioda S Matsuda K Purification and properties of ghr*elin from the intestin *e of the goldfish, *Carassius auratus* Peptides 2009 30 758 765 10.1016/j.peptides.2008.12.016 19150635

[B13] Kaiya H Kojima M Hosoda H Koda A Yamamoto K Kitajima Y Matsumoto M Minamitake Y Kikuyama S Kangawa K *Bullfrog ghrelin is modified *by n-octanoic acid at its third threonine residue J Biol Chem 2001 276 40441 40448 10.1074/jbc.M105212200 11546772

[B14] Kaiya H Geyten S Van der Kojima M Hosoda H Kitajima Y Matsumoto M Geelissen S Darras VM Kangawa K Chicken ghrelin: purification cDNA cloning and biological activity Endocrinology 2002 143 3454 3463 10.1210/en.2002-220255 12193558

[B15] Unniappan S Lin X Cervini L Rivier J Kaiya H Kangawa K Peter RE Goldfish ghelin: molecular characterization of the complementary deoxyribonucleic acid, partial gene structure and evidence for its stimulatory role in food intake Endocrinology 2002 143 4143 4146 10.1210/en.2002-220644 12239128

[B16] Matsuda K Miura T Kaiya H Maruyama K Shimakura S Uchiyama M Kangawa K Shioda S Regulation of food intake by acyl and des-acyl ghrelins in the goldfish Peptides 2006 27 2321 2325 10.1016/j.peptides.2006.03.028 16687192

[B17] Miura T Maruyama K Shimakura S Kaiya H Uchiyama M Kangawa K Shioda S Matsuda K Regulation of food intake in the goldfish by interaction between ghrelin and orexin Peptides 2007 28 1207 1213 10.1016/j.peptides.2007.03.023 17481778

[B18] Riley LG Fox BK Kaiya H Hirano T Grau EG Long-term treatment of ghrelin stimulates feeding, fat deposition, and alters the GH/IGF-I axis in the tilapia, *Oreochromis mossambicus* Gen Comp Endocrinol 2005 142 234 240 10.1016/j.ygcen.2005.01.009 15862568

[B19] Shepherd BS Johnson JK Silverstein JT Parhar IS Vijayan MM McGuire A Weber GM Endocrine and orexigenic actions of growth hormone secretagogues in rainbow trout (*Oncorhynchus mykiss*) Comp Biochem Physiol A 2007 146 390 399 10.1016/j.cbpa.2006.11.004 17240179

[B20] Furuse M Tachibana T Ohgushi A Ando R Yoshimatsu T Denbow DM Intracerebroventricular injection of ghrelin and growth hormone releasing factor inhibits food intake in neonatal chicks Neurosci Lett 2001 30 123 126 10.1016/S0304-3940(01)01621-4 11248438

[B21] Saito ES Kaiya H Tachibanat T Tomonaga S Denbow DM Kangawa K Furuse M Inhibitory effect of ghrelin on food intake is mediated by the corticotropin-releasing factor system in neonatal chicks Regul Pept 2005 125 201 208 10.1016/j.regpep.2004.09.003 15582733

[B22] Shousha S Nakahara K Kojima M Miyazato M Hosoda H Kangawa K Murakami N Different effects of peripheral and central ghrelin on regulation of food intake in the Japanese quail Gen Comp Endocrinol 2005 141 178 183 10.1016/j.ygcen.2004.12.021 15748719

[B23] Geelissen SM Swennen Q Geyten SV Kühn ER Kaiya H Kangawa K Decuypere E Buyse J Darras VM Peripheral ghrelin reduces food intake and respiratory quotient in chicken Domest Anim Endocrinol 2006 30 108 116 10.1016/j.domaniend.2005.06.005 16054797

[B24] Tachibana T Kaiya H Denbow DM Kangawa K Furuse M Central ghrelin acts as an anti-dipsogenic peptide in chicks Neurosci Lett 2006 405 241 245 10.1016/j.neulet.2006.07.019 16901639

[B25] Kawakoshi A Kaiya H Riley LG Hirano T Grau EG Miyazato M Hosoda H Kangawa K Identification of a ghrelin-like peptide in two species of shark, *Sphyrna lewini *and *Carcharhinus melanopterus* Gen Comp Endocrinol 2007 151 259 268 10.1016/j.ygcen.2006.10.012 17362948

[B26] Kaiya H Kojima M Hosoda H Moriyama S Takahashi A Kawauchi H Kangawa K Peptide purification, cDNA and genomic DNA cloning, and functional characterization of ghrelin in rainbow trout Endocrinology 2003 144 5215 5226 10.1210/en.2003-1085 12970156

[B27] Bustin SA Quantification of mRNA using real-time reverse transcription PCR (RT-PCR): trends and problems J Mol Endocrinol 2002 29 23 39 10.1677/jme.0.0290023 12200227

[B28] Sasayama Y Suzuki N Oguro C Takei Y Takahashi A Watanabe TX Nakajima K Sakakibara S Calcitonin of the stingray: comparison of the hypocalcemic activity with other calcitonins Gen Comp Endocrinol 1992 86 269 274 10.1016/0016-6480(92)90110-6 1601276

[B29] Büllesbach EE Schwabe C Lacy ER Identification of a glycosylated relaxin-like molecule from the male Atlantic stingray, Dasyatis sabina Biochemistry 1997 36 10735 10741 10.1021/bi970393n 9271504

[B30] Matsuda K Yoshida T Nagano Y Kashimoto K Yatohgo T Shimomura H Shioda S Arimura A Uchiyama M Purification and primary structure of pituitary adenylate cyclase activating polypeptide (PACAP) from the brain of an elasmobranch, stingray, *Dasyatis akajei* Peptides 1998 19 1489 1495 10.1016/S0196-9781(98)00091-6 9864054

[B31] Amemiya Y Takahashi A Suzuki N Sasayama Y Kawauchi H Molecular cloning of proopiomelanocortin cDNA from an elasmobranch, the stingray, *Dasyatis akajei* Gen Comp Endocrinol 2000 118 105 12 10.1006/gcen.1999.7444 10753572

[B32] Yang J Brown MS Liang G Grishin NV Goldstein JL Identification of the acyltransferase that octanoylates ghrelin, an appetite-stimulating peptide hormone Cell 2008 132 I387 396 10.1016/j.cell.2008.01.017 18267071

[B33] Gutierrez JA Solenberg PJ Perkins DR Willency JA Knierman MD Jin Z Witcher DR Luo S Onyia JE Hale JE Ghrelin octanoylation mediated by an orphan lipid transferase Proc Natl Acad Sci USA 2008 105 6320 6325 10.1073/pnas.0800708105 18443287 PMC2359796

[B34] Nishi Y Hiejima H Hosoda H Kaiya H Mori K Fukue Y Yanase T Nawata H Kangawa K Kojima M Ingested medium-chain fatty acids are directly utilized for the acyl-modification of ghrelin Endocrinology 2005 146 2255 2264 10.1210/en.2004-0695 15677766

[B35] Yamato M Sakata I Wada R Kaiya H Sakai T Exogenous administration of octanoic acid accelerates octanoylated ghrelin production in the proventriculus of neonatal chicks Biochem Biophys Res Commun 2005 333 583 589 10.1016/j.bbrc.2005.05.107 15953586

[B36] Piek T Hue B Le Corronc H Mantel P Gobbo M Rocchi R Presynaptic block of transmission in the insect cns by mono- and di-galactosyl analogues of vespulakinin 1, a wasp (Paravespula maculifrons) venom neurotoxin Comp Biochem Physiol C 1993 105 189 196 10.1016/0742-8413(93)90193-O 8103727

[B37] Bousfield GR Butnev VY Gotschall RR Baker VL Moore WT Structural features of mammalian gonadotropins Mol Cell Endocrinol 1996 125 3 19 10.1016/S0303-7207(96)03945-7 9027339

[B38] Wells L Hart GW O-GlcNAc turns twenty: functional implications for post-translational modification of nuclear and cytosolic proteins with a sugar FEBS Lett 2003 546 154 158 10.1016/S0014-5793(03)00641-0 12829252

[B39] Semenov AG Postnikov AB Tamm NN Seferian KR Karpova NS Bloshchitsyna MN Koshkina EV Krasnoselsky MI Serebryanaya DV Katrukha AG Processing of pro-brain natriubetic peptide is suppressed by O-Glycosylation in the region close to the cleavage site Clin Chem 2009 55 489 498 10.1373/clinchem.2008.113373 19168558

[B40] Torsello A Ghe' C Bresciani E Catapano F Ghigo E Deghenghi R Locatelli V Muccioli G Short ghrelin peptides neither displace ghrelin binding in vitro nor stimulate GH release in vivo Endocrinology 2002 143 1968 1971 10.1210/en.143.5.1968 11956180

[B41] Caminos JE Gualillo O Lago F Otero M Blanco M Gallego R Garcia-Caballero T Goldring MB Casanueva FF Gomez-Reino JJ Dieguez C The endogenous growth hormone secretagogue (ghrelin) is synthesized and secreted by chondrocytes Endocrinology 2005 146 1285 1292 10.1210/en.2004-1379 15576457

[B42] Nelson JS Fishes of the world 2006 4 John Wiley & Sons, Inc., New York 601

[B43] McEachran JD Aschliman N Carrier JC, Musick JA, Heithaus MR Phylogeny of Batoidea Biology of sharks and their relatives 2004 Boca Raton, CRC Press 79 114

[B44] Douady CJ Dosay M Shivji MS Stanhope MJ Molecular phylogenetic evidence refuting the hypothesis of Batoidea (rays and skates) as derived sharks Mol Phylogen Evol 2003 26 215 221 10.1016/S1055-7903(02)00333-0 12565032

[B45] Zhang JV Ren PG Avsian-Kretchmer O Luo CW Rauch R Klein C Hsueh AJ Obestatin, a peptide encoded by the ghrelin gene, opposes ghrelin's effects on food intake Science 2005 310 996 999 10.1126/science.1117255 16284174

